# Snapshot of Phylogenetic Groups, Virulence, and Resistance Markers in* Escherichia coli* Uropathogenic Strains Isolated from Outpatients with Urinary Tract Infections in Bucharest, Romania

**DOI:** 10.1155/2019/5712371

**Published:** 2019-05-20

**Authors:** Violeta Corina Cristea, Irina Gheorghe, Ilda Czobor Barbu, Laura Ioana Popa, Bogdan Ispas, Georgiana Alexandra Grigore, Irina Bucatariu, Gabriela Loredana Popa, Maria-Cristina Angelescu, Alexandra Velican, Luminita Marutescu, Marcela Popa, Mariana Carmen Chifiriuc, Ioan Mircea Popa

**Affiliations:** ^1^Central Laboratory Synevo-Medicover, Bucharest, Romania; ^2^University of Medicine and Pharmacy Carol Davila, Bucharest, Romania; ^3^Department of Microbiology and Immunology, Faculty of Biology, University of Bucharest, Romania; ^4^Research Institute of the University of Bucharest (ICUB), Bucharest, Romania; ^5^National Medico-Military Institute for Research and Development Cantacuzino, Bucharest, Romania

## Abstract

**Background:**

Urinary tract infections (UTIs) caused by Uropathogenic* Escherichia coli* (UPEC) are among the most common infections worldwide, including Romania. To the best of our knowledge, this is the first study performed on a significant number of community-acquired (CA) UPEC strains isolated from Romanian outpatients, aiming to evaluate and establish potential correlations among the phylogenetic groups (PG), resistance profiles, and the virulence factors (VF) genes of the CA-UPEC isolates.

**Materials/Methods:**

The present study was performed on a total of 787 UPEC nonrepetitive isolates consecutively isolated during one month from outpatients with CA-UTIs, visiting one of the biggest laboratories in Bucharest, Romania, receiving patients from all over the country. The strains identification was performed by MALDI TOF and the susceptibility patterns were tested using Microscan according to CLSI guidelines. PCR assays were performed to detect the presence of different VFs (*fimH* gene encoding for type 1 fimbriae,* afaBC* for A fimbriae,* sfaDE* for S fimbriae,* KpsMTII* for capsule,* hlyA* for haemolysin A,* hlyD* for haemolysin D, and* cnf-1* for tumor necrosis factor), the phylogenetic groups (PG) A, B1, B2, and D, and the extended spectrum beta-lactamases (ESBLs) genes.

**Results:**

The 787 CA-UPEC strains were isolated predominantly from female patients (90.95%) of >30 years (~74%). The resistance rates were 47.52% for ampicillin, 41.16% for tetracycline, 24.39% for cotrimoxazole, 19.18% for amoxicillin-clavulanic acid, 15.50% for cefazolin, 14.99% for ciprofloxacin, and 14.86% for levofloxacin; 35.19% of the investigated strains were MDR and 9.03% ESBL producers (from which 42.25% were positive for* bla*CTX-M, 38.02% for* bla*TEM, and 19.71% for* bla*SHV).* FimH *was the most frequent virulence gene (93.90%) followed by* hlyD* (44.34%);* afaBC* (38.24%);* KpsMTII* (32.65%); sfaDE (23.88%);* hlyA* (12.45%); and* cnf-1* (7.75%). The distribution of the analyzed UPEC strains in phylogenetic groups was different for non-MDR and MDR strains. Overall, 35% of the strains belonged to the phylogenetic group B2 (harboring the* yjaA* gene); 27% to group B1 (confirmed by the presence of the* TspE4C2 *fragment); 16% to group D; and 22% to group A. The CA-UPEC strains included in PG B1 and PG B2 proved to be the most virulent ones, the number of strains carrying multiple VFs (>3) being significantly larger as compared to strains belonging to PG A and PG D) (p<0,0001). The presence of one or two ESBL genes was significantly associated (p =0.0024) with PGs A and D.

**Conclusions:**

Our findings showed that the community UPEC strains circulating in Bucharest, Romania, belong predominantly to group B2 and >90% harbored the* fimH* gene. High MDR resistance rates were observed, as well as extended VF profiles, highlighting the importance of this type of studies for improving the epidemiological surveillance and the therapeutic or prophylactic management of the respective infections, in the context of antibiotic resistance emergence.

## 1. Background

Urinary tract infection (UTI) caused by Uropathogenic* Escherichia coli* (UPEC) is the most common community-acquired (CA) and nosocomial infection [1, 2] and represents an important worldwide health problem, leading to considerable morbidity costs [3–5].* E. coli* is the leading cause of UTIs, being responsible for 75-90% of UTIs in ambulatory patients [6]. These isolates encode different virulence factors (VFs), like toxins, capsules, invasins, and adhesins, which are contributing to the UPEC strains pathogenicity and consequently, to the severity of the produced UTI [7]. Moreover, the emergence of multidrug-resistant (MDR) UPEC strains is currently leading to major difficulties in treating the infected patients [8–10].

UPEC strains have been classified into several main phylogenetic groups (PG) (A, B1, B2, C, D, E, and F) and one* Escherichia* cryptic clade I, based on the combination of four genetic markers:* arpA, chuA, yjaA*, and the DNA fragment* TspE4C2 *[11]. UPEC strains usually belong to group B2 and to a lesser extent, to group D, whereas commensal strains belong to groups A and B1 [12]. Among B2 strains,* E. coli* sequence type 131 (ST131) is considered an important emerging pathogen, harboring numerous resistance and VF genes [13]. Strains belonging to this group are resistant to most *β*-lactam antibiotics, mediated by the production of extended spectrum *β*-lactamases (ESBLs). ESBLs are plasmid-encoded enzymes which confer resistance to penicillins, broad-spectrum cephalosporins, and monobactams, but not to cephamycins and carbapenems. Moreover it has been revealed that ESBL-producing isolates show coresistance to aminoglycosides, quinolones, tetracyclines, nitrofurantoin, and trimethoprim-sulfamethoxazole [14]. The MDR phenotype is due to the presence of large plasmids, which commonly carry resistance genes for *β*-lactams, quinolones, aminoglycosides, and cotrimoxazole. The most common *β*-lactamases in* E. coli* strains are TEM, SHV, and CTX-M types [14]. Most ST131 strains belong to the O25:H4 serotype, with the specific O25b type. However, ST131 strains with serotype O16:H5 have been recently identified, as well as some others that are nontypeable for O and H antigens [15]. UPEC clones ST69, ST95, and ST73 are also frequent causes of UTIs and bloodstream infections. A study performed in Brazil showed that UTIs in men were more frequently caused by PG B2 isolates, harboring an extended VFs genes profile [12]. A study performed in Iran on 232 UPEC strains revealed that the most frequent PG was D (both for hospital and CA infections (58%), exhibiting the highest number of VF and resistance markers [16].

The aim of the study was to characterize the resistance and virulence profiles of recently isolated UPEC strains from outpatients visiting Synevo Central Laboratory, Medicover, in Bucharest, south Romania, and to establish potential correlations among PG, resistance, and VF genes profiles of the analyzed strains. To the best of our knowledge, this is the first study performed on a significant number of UPEC strains isolated from CA-UTIs in Romania.

## 2. Results and Discussion

The 787 UPEC strains were isolated predominantly from female patients (90.95%) and the distribution on age groups was the following: 0-16 years (8.66%); 16-30 years (16.94%); 30-50 years (35.79%), and 50-90 years (38.29%).

### 2.1. Phylogenetic Group Distribution of UPEC Strains

The PG analysis of the UPEC strains [17, 18] showed that 35% of the strains belonged to B2 (harboring the* yjaA* gene); 27% to B1 (confirmed by the presence of* TspE4 C2* gene); 16% to D and 22% to A. The obtained results are very close to those reported from Nüesch-Inderbinen et al., in UPEC strains isolated from community-acquired UTI in Switzerland [19].

### 2.2. Antibiotic Resistance Profiles of UPEC Strains

In this study, the prevalence of antimicrobial resistance markers was relatively high for drugs commonly used as emergency therapy in the treatment of UTIs, such as ampicillin (47.52%), tetracycline (41.16%), trimethoprim-sulfamethoxazole (cotrimoxazole) (24.39%), amoxicillin-clavulanic acid (19.18%), cefazolin (15.50%), and fluoroquinolones (14.99% for ciprofloxacin and 14.86% for levofloxacin). Moreover, 35.19% of the UPEC strains were MDR, according to Magiorakos et al., 2012 [20] criteria. Lower resistance percentages were recorded for aztreonam, cefepime, ceftriaxone, piperacillin-tazobactam, and gentamicin (Table 1), in contrast with the study performed by Lavigne et al., in 2016, which reported higher resistance percentages for amoxicillin, amoxicillin-clavulanic acid, nalidixic acid, ciprofloxacin, and nitrofurantoin and for cotrimoxazole in* E. coli *strains from CA-UTI.

Out of the total of the analyzed strains, 71 isolates (9.03%) were resistant to third-generation cephalosporins, all of them being positive for the investigated ESBL genes, as follows:* bla*CTX-M (42.25%) and* bla*TEM (38.02%) and for* bla*SHV (19.71%) (Table 2). Regarding the resistance profiles of the ESBL strains, they were highly resistant to ampicillin (100%), aztreonam (98.59%), cefepime (98.59%), ceftriaxone (91.59%), cefazolin (87.32%), tetracycline (73.23%), ciprofloxacin (71.83%), and levofloxacin (70.42%) (Table 2).

A study performed in Algeria, aiming to investigate antibiotic resistance and VF in 150 nonrepetitive CA-UPEC isolates has revealed a MDR rate of 46.7% [21]. The detected* bla* genes were* bla*TEM (96.8% of amoxicillin-resistant isolates),* bla*CTX-M-15 (4%),* bla*AmpC (4%),* bla*SHV-2a,* bla*TEM-4,* bla*TEM-31, and* bla*TEM-35 (0.7%) [21].

**Figure 1:**
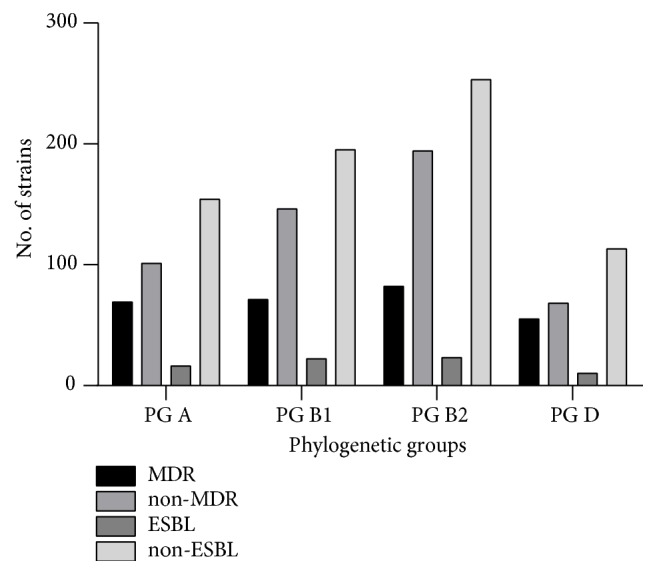
The distribution of ESBL and MDR isolates by phylogenetic groups.

Our study has revealed that the MDR strains were classified in PG D (44.71%), followed by PG A (40.58%), PG B1 (32.71%), and PG B2 (29.71%), while the non-MDR strains were predominantly associated with PG B2, followed by B1, and to a lesser extent with the other two PGs (Figure 1). A different distribution was recorded for the ESBL-producing strains, which belonged to PG B1 (10.13%); PG A (9.41%); PG B2 (8.33%), and PG D (8.13%). However, the statistical analyses did not reveal any statistical significance of the correlation between the antibiotic resistance and* E. coli *phylogenetic groups.

In other studies, the CA-UPEC isolates belonged to phylogroups B2+D (50%), A+B1 (36%), and F+C+Clade I (13%). Most of D (72.2%) and 38.6% of B2 isolates were MDR and harbored the most extended VFs profiles [21].

### 2.3. Virulence Profiles of UPEC Strains

Regarding the virulence markers detected in the analyzed UPEC strains, the fimH gene was the most encountered VF (93.90%) followed by* hlyD* (44.34%);* afaBC* (38.24%);* KpsMTII* (32.65%);* sfaDE* (23.88%);* hlyA* (12.45%); and* cnf-1* (7.75%) (Table 3).

**Figure 2:**
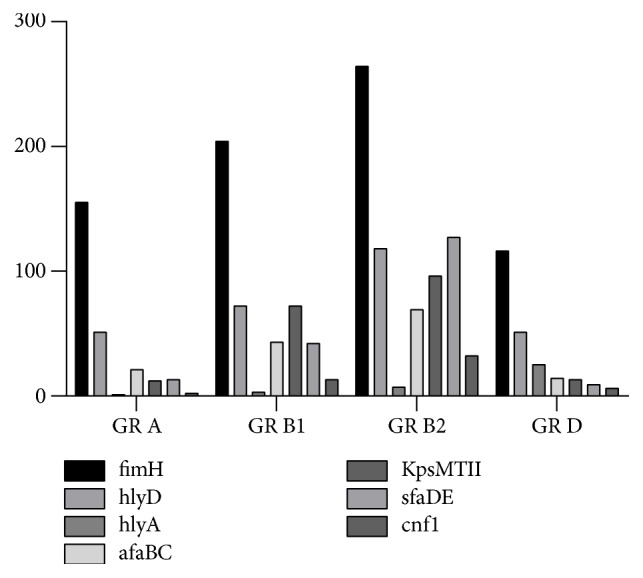
The distribution of VFs by PGs.

Distribution of the analyzed strains harboring different VF in PGs revealed a relatively equal distribution among the PG for fimH, while others (*hlyA, afaBC, kpsMTII, sfaDE, *and* cnf-1*) were significantly associated with certain PGs (Figure 2 and Table 3).

Regarding the correlation between the pathogenicity level (the number of VF genes) and the phylogenetic groups, the strains belonging to PG D (n=123 strains) revealed the following VF genes profiles: 5 VFs in 4.06% of the investigated strains; 4 VFs (*fimH, hlyD, sfaDE cnf-1, *and* hlyA*) in 5.69% of the isolates; 3 VFs (*fimH* was present in all the combinations also) in 16.26%; 2 VFs in 36.58%; and one VFs (37.39% of the isolates). The strains belonging to the PG A (n=170 strains) revealed 4 VFs (*fimH, hlyD, sfaDE, *and* cnf-*1) in 1.76% of the isolates; 3 VFs (fimH in all the combinations and* hlyD/afaBC/kpsMTII/sfaDE/cnf-1*) in 7.64%; 2 VFs in 32.94%; and one VFs (54.11% from which* fimH *was revealed by 96.73% and* hlyD *from 3.26% of the isolates). In case of PG B1 strains there were detected up to 5 VFs (*fimH, hlyD, kpsMTII, sfaDE, cnf-1 *or*hlyA*) in 1.84% of the isolates; 4 VFs (with* fimH *being revealed in all of them excepting one) in 8.29% of the isolates; 3 VFs in 20.27%; 2 VFs in 35.48%; and one VFs (32.71% of the isolates). The strains classified in PG B2 demonstrated the presence of 6 VFs in 0.36% of the UPEC isolates; 5 VFs in 5.79%; 4 VFs (*fimH* being revealed in all of them) in 18.47% of the isolates; 3 VFs in 25.36%; 2 VFs in 28.62%; and one VF in 19.92% of the isolates.

**Figure 3:**
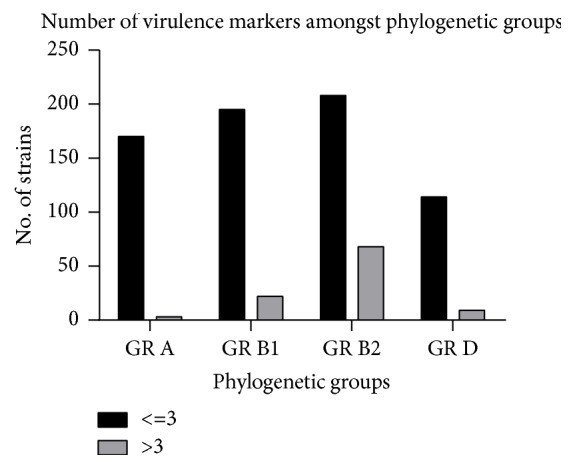
The distribution of the number of VFs among PGs.

The strains classified in PG B1 and PG B2 were the most virulent ones, the number of strains carrying >3VFs being significantly larger than that of strains belonging to PG A and PG D, where predominating the strains carrying less VFs (≤ 3) (p<0.0001) (Figure 3).

Only few studies are reported in literature for CA-UPEC isolates. A study performed on Uruguayan children with UTIs revealed that 48.2% of the* E. coli* isolates belonged to PG D and 35.5% to PG B2, with the most frequent VFs being kpsMTII and fimH [22]. Among 59 isolates of UPEC isolated in Pakistan from CA-UTIs, the PG B2 was the most frequent (50%), followed by PG A, B1 (19% each), and D (12%). Isolates present in group D showed the highest number of VFs, among which the most frequent were hlyA (37%), sfaDE (27%), papC (24%), cnf1 (20%), eaeA (19%), and afaBC3 (14%) [23].

Of the UPEC adhesins, fimH, a type 1 fimbriae, has a crucial role in UPEC colonization in the bladder, which is required for the initiation of UTI [24].* E. coli* afimbrial adhesin (Afa) encoded by* afa* gene has been reported in cases of pyelonephritis and recurring cystitis; another adhesin that acts as a virulence factor is S fimbrial adhesin, which is encoded by* sfa* genes [25]. Other very important virulence factors in UPEC strains, like toxins, mediating invasion, dissemination, and persistence of bacteria in host cells have been demonstrated [26]. The most important soluble virulence factor is *α*-hemolysin (HlyA), which is encoded by the* hly* gene. Also, the cytotoxic necrotizing factor 1 (CNF1) is one of the most important virulence factors of* E. coli* involved in the development of an UTI. It has been revealed that *α*-hemolysin and CNF1 mediate the release of iron from red blood cells, induce dysfunction of phagocytic cells, and exhibit direct cytotoxicity to the tissues [27]. Some authors report that the prevalence ratios of* sfa*,* hlyA,* and iron uptake genes were 2.2 to 3.5 times more prevalent among outpatients compared with inpatients UTI isolates [28].

According to the present observations,* fimH* gene had the highest frequency (93.90%) among the tested VF genes, while* cnf-1* had the lowest one (7.75%) (Table 4). Similar results have been revealed in Romania by Grosu et al. in 2017 [29] in* E. coli* strains isolated from the ambulatory sector of Central Laboratory Regina Maria hospital in Bucharest. The* fimH* gene was also reported to have a high prevalence (>90%-100%) among UPEC strains isolated in other countries, and some authors are stating that FimH could be used as a possible diagnostic marker and/or vaccine candidate [30–32]. The study of Tobasi et al. [28] performed on 156 UPEC isolated from symptomatic and asymptomatic UTI outpatients and inpatients revealed that* fimH* was present in all analyzed strains. Derakhshandeh et al., in 2015 [33], have reported that, from 85 UPEC clinical isolates, 65.9% belonged to phylogenetic group A, 17.6% belonged to B2, and 16.5% of the isolates were found to belong to group D;* fimH *has also been reported with the highest frequency among the tested VF genes, while* cnf-1* had the lowest one, similar to our results.

According to Rodriguez-Siek study in 2005 [34], most of UPEC causing UTI in human revealed capsule, the capsular antigen K1 being more often observed in UPEC. The capsule production in* E. coli *trains is mediated by*kpsMT* (encoding for K1 antigen) and* kpsMTII *genes [35]. A study performed on a total of 194* E. coli *strains isolated in Mexico from CA-UTIs has shown that* kpsMT* was the most frequently occurring virulence gene among the UPEC strains (92.2% strains), the* fim* gene being also recorded with a high positivity rate (61.3%) [36]. Farajzadah et al., in 2018 [16], have reported that, among the 232 analyzed UPEC strains, the most frequently encountered PG was D (58%) responsible for majority of nosocomial (64.7%) and community (48.4%) acquired infections with the largest panel of VF genes, including* kpsMT *(23%) and* cnf* (29.6%). Ochoa et al., in 2016 [37], have found that, among 500 UPEC clinical strains, 103 were MDR-UPEC strains and mainly associated with the phylogenetic groups D (54.87%) and B2 (39.02%) with a high percentage of positivity for* fimH*, an iron uptake gene (*chuA*), and a toxin gene (*hlyA*).

We have also investigated the potential correlations between the PG and VF genes and the presence of different resistance phenotypes (multiple logistic regression) or ESBL genes (chi square). Our results pointed out that there is no statistically significant correlation between PG and VF and the presence of certain resistance phenotypes, which suggests that there are no particular clones associated with UTIs in Romania. On the other hand, the presence of one or two ESBL genes was significantly associated (p =0.0024) with PGs A and D.

In France, UPEC isolated from UTIs belonged more frequently to phylotypes B2 and D, the strains susceptible to ciprofloxacin harboring specific VFs profiles, more extended in comparison with the ciprofloxacin-resistant strains [38]. Another study performed on 146* E. coli* strains isolated from cystitis and pyelonephritis in Turkey investigated the relationship among PGs and various adhesion virulence genes. The* sfa/focDE* genes were more frequent in ampicillin, amikacin, gentamicin, nalidixic acid, norfloxacin, cefuroxime, ceftriaxone, cefazolin, cefotaxime, ciprofloxacin and cotrimoxazole susceptible and extended spectrum *β*-lactamase (ESBL), and multidrug resistance (MDR) negative isolates.* fimH* was more common in amoxicillin-clavulanic acid susceptible isolates. The* afa* gene was more frequent in resistant isolates than in susceptible ones [39].

## 3. Conclusions

To the best of our knowledge, this is the first study performed on a significant number of* E. coli *strains isolated from outpatients with community-acquired urinary tract infections in Bucharest, Romania, aiming to investigate the correlations among the phylogenetic group, resistance, and virulence profiles of CA-UPEC strains. The analyzed strains exhibited resistance rates ranging from 47.52% for ampicillin to 14.86% for levofloxacin, 35.19% were MDR phenotype, and 9.03% were ESBL producers. The* fimH* gene was the most frequent (93.90%), followed by* hlyD* (44.34%);* afaBC *(38.24%);* KpsMTII* (32.65%);* sfaDE* (23.88%);* hlyA*(12.45%); and* cnf-1* (7.75%). The phylogenetic group distribution was different, depending on the resistance phenotype. Overall, our findings showed that the CA-UPEC strains isolated from outpatients in Bucharest, Romania, belong predominantly to group B2 and >90% harbor the* fimH* gene. High MDR resistance rates were observed, the ESBL phenotype being associated with PGs A and D. The most extended VF profiles were encountered in CA-UPEC strains classified in the PGs B1 and B2. The obtained results highlight the importance of this type of studies for improving the epidemiological surveillance and the therapeutic or prophylactic management of the respective infections, in the context of antibiotic resistance emergence.

## 4. Methods

The study was conducted on a total of 787 strains isolated during one month in 2018 from outpatients visiting Synevo Central Laboratory, Medicover, in Bucharest, Romania. The strains identification was performed using the MALDI TOF system and the susceptibility patterns were tested by Microscan according to CLSI 2018 guidelines.

### 4.1. DNA Extraction and Molecular Detection

The genetic support of the antibiotic resistance (ESBLs) and virulence markers was investigated by simplex and multiplex PCR, using a reaction mix of 20*μ*l (PCR Master Mix 2x, Thermo Scientific containing MgCl_2_ 1.2mM, dNTP 2*μ*M DNA 0.2U Taq-pol 1x and Reaction buffer until the final volume) to which the primers at 0.5*μ*M and and 1 *μ*l of bacterial DNA extracted by an adapted alkaline extraction method. In this purpose, 1-5 colonies of bacterial cultures were suspended in 1.5 ml tubes containing 20 *μ*l solution of 0.05M NaOH (sodium hydroxide) and 0.25% SDS (sodium dodecyl sulphate). The amplification program was conducted under the following conditions: 94°C,10 min; 94°C, 30s; 52°C, 40s, 36 cycles; 72°C 50s; 72°C 5 min.

Bacterial DNA were subjected to simplex PCR targeting the chuA gene, the yjaA gene, and an unspecified DNA fragment termed TspE4.C2, as described previously [17]. Isolates were classified as belonging to one of the four phylogenetic groups A, B1, B2, or D. The sequence of the primers used in PCR experiments, the amplicon size obtained and the sources are presented in Tables 5 and 6.

## 5. Statistical Analysis

Statistical analysis was carried out using chi square and chi square test for trend tests using Graph Pad Prism version 8.0.1 (244) and multiple logistic regression using Stats Direct version 3. For all statistical tests, p values <0.05 were considered significant.

## Figures and Tables

**Table 1:** Antibiotic resistance phenotypes of *E. coli *strains isolated from CA-UTIs in Romania.

Antibiotic	GR A	GR B1	GR B2	GR D	Number (percentage)
AMP	90 (52.94%)	98 (45.16%)	125 (45.28%)	66 (53.65%)	374 (47.52%)
TET	75 (44.11%)	91 (41.93%)	98 (35.50%)	61 (49.59%)	324 (41.16%)
SXT	43 (25.29%)	52 (23.96%)	60 (21.73%)	41 (33.33%)	192 (24.39%)
AMC	38 (22.35%)	41 (18.89%)	55 (19.92%)	24 (19.51%)	151 (19.18%)
CFZ	35 (20.58%)	29 (13.36%)	42 (15.21%)	17 (13.82%)	122 (15.50%)
CIP	34 (20%)	38 (17.51%)	28 (10.14%)	18 (14.63%)	118 (14.99%)
LEV	34 (20%)	38 (17.51%)	28 (10.14%)	18 (14.63%)	117 (14.86%)
ATM	16 (9.41%)	21 (9.67%)	27 (9.78%)	11 (8.94%)	79 (10.03%)
FEP	16 (9.41%)	21 (9.67%)	26 (9.42%)	10 (8.13%)	73 (9.27%)
CRO	16 (9.41%)	21 (9.67%)	26 (9.42%)	10 (8.13%)	72 (9.14%)
TZP	17 (10%)	11 (5.06%)	16 (5.79%)	3 (2.43%)	47 (5.97%)
CN	12 (7.05)	6 (2.76%)	10 (3.62%)	7 (5.69%)	34 (4.32%)
NIT	5 (2.94%)	8 (3.68%)	7 (2.53%)	4 (3.25%)	24 (3.04%)
IMP	0	0	0	1 (0.81%)	1 (0.12%)
MEM	0	0	0	0	0
ETP	0	0	0	0	0

AMP, ampicillin; TET, tetracycline; SXT, trimethoprim-sulfamethoxazole; AMC, amoxicillin-clavulanic acid; CFZ, cefazolin; CIP, ciprofloxacin; LEV, levofloxacin; ATM, aztreonam; FEP, cefepime; CRO, ceftriaxone; TZP, piperacillin-tazobactam; CN, gentamicin; NIT, nitrofurantoin; IMP, imipenem; MEM, meropenem; ETP, ertapenem.

**Table 2:** Antibiotic resistance phenotypes of ESBL strains.

Antibiotic	Number (percentage)
AMP	71 (100%)
ATM	70 (98.59%)
FEP	70 (98.59%)
CRO	65 (91.59%)
CFZ	62 (87.32%)
TET	52 (73.23%)
CIP	51 (71.83%)
LEV	50 (70.42%)
SXT	34 (47.89%)
AMC	32 (45.07%)
TZP	17 (23.94%)
NIT	6 (8.45%)

**Table 3:** The association of different VF genes with PGs.

	GR A	GR B1	GR B2	GR D	p-value
*fimH*	155 (91.17%)	204 (94%)	264 (95.65%)	116 (94.30%)	0.2873
*hlyD*	51 (30%)	72 (33.17%)	118 (42.75%)	51 (41.46%)	0.0196
*hlyA*	1 (0.58%)	3 (1.38%)	7 (2.53%)	25 (20.32%)	0.0693
*afaBC*	21 (12.35%)	43 (19.81%)	69 (25%)	14 (11.38%)	0.0010
*KpsMTII*	12 (7.05%)	72 (33.17%)	96 (34.78%)	13 (10.56%)	<0.0001
*sfaDE*	13 (7.64%)	42 (19.35%)	127 (46.01%)	9 (7.31%)	<0.0001
*cnf-1*	2 (1.17%)	13 (5.99%)	32 (11.59%)	6 (4.87%)	0.0005

**Table 4:** Virulence genes profiles and PGs in the investigated *E. coli *strains.

VF	Number (percentage)
*fimH*	739 (93.90%)
*hlyD*	349 (44.34%)
*afaBC*	301 (38.24%)
*kpsMTII*	257 (32.65%)
*sfaDE*	188 (23.88%)
*hlyA*	98 (12.45%)
*cnf-1*	61 (7.75%)
*PGs*	
*B2*	276 (35%)
*B1*	216 (27%)
*A*	173 (22%)
*D*	122 (16%)

**Table 5:** Primers sequences used in simplex and multiplex PCR assays for genes encoding BLSE.

The gene	Primer	Nucleotide sequence	Amplification size	References
*bla*_TEM_	TEM-F TEM-R	5'-ATGAGTTTTCAACATTTTCG-3' 5'-TTACCAATGCTTAATCAG TG-3'	861	Eftekar et al., 2005 [40]

*bla*_SHV_	SHV-F SHV-R	5'-GCCCTCACTCAAGGATGTAT-3' 5'-TTAGCGTTGCCAGTGCTCGA-3'	888	Naas et al., 1999 [41]

*bla*_CTX-M_	CTX-M-F CTX-M-R	5'-CGCTGTTGTTAGGAAGTGTG-3' 5'-GGCTGGGTGAAGTAAGTGAC-3'	730	Israil et al., 2013 [42]

Table 5 is partially reproduced from Grosu et al. 2017 [underthe Creative Commons Attribution License/public domain].

**Table 6:** Primers sequences used in simplex and multiplex PCR assays for virulence genes.

The gene	Primer	Amplification size and Tm	References
*chuA*	F: 5′-GACGAACCAACGGTCAGGAT-3′ R: 5′-TGCCGCCAGTACCAAAGACA-3′	279 bp (multiplex) 55°C	Clermont et al., 2000

*yjaA*	F: 5′-TGAAGTGTCAGGAGACGCTG-3′ R: 5′-ATGGAGAATGCGTTCCTCAAC-3′	211bp (multiplex) 55°C	Clermont et al., 2000

*TspE4C2*	F: 5′-GAGTAATGTCGGGGCATTCA-3′ R:5′-CGCGCCAACAAAGTATTACG-3′	152 bp (multiplex) 55°C	Clermont et al., 2000

*hlyD*	F: 5′- CTCCGGTACGTGAAAAGGAC-3′ R: 5′-GCCCTGATTACTGAAGCCTG-3′	904 bp 55°C	Rodrigues-Sike et al., 2005

*kpsMTII*	F: 5′- GCG CAT TTG CTG ATA CTG TTG-3′ R: 5′-CAT CAG ACG ATA AGC ATG AGC A-3′	272 bp 60°C	Johnson et al., 2005 [43]

*hlyA*	F:5′-AACAAGGATAAGCACTGT TCTGGC T-3′ R:5′-ACCATATAAGCGGTCATT CCC GTC A-3′	1,177 bp 60°C	Yamamoto et al., 1995 [44]

*sfaD/E*	F:5′-CGGAGGAGTAATTACAAACCTGGCA -3′ R: 5′- CTCCGGAGAACTGGGTG ATCTTA C-3′	408 bp 60°C	Blanco et al., 1997 [45]

*fimH*	F: 5′-TGC AGA ACG GAT AAG CCG TGG -3′ R: 5′- GCA GTC ACC TGC CCT CCG GTA -3′	508 bp 63°C	Rodrigues-Sike et al., 2005

*afaBC*	F: 5′-GCTGGGCAGCAAACTGATAACTCTC -3′ R:5′CATCAAGCTGTTTGTTCGTCCGCCG-3	793 bp 63°C	Blanco et al., 1997

*cnf-1*	F: 5′- GAA CTT ATT AAG GAT AGT-3′ R: 5′-CAT TAT TTA TAA CGC TG-3′	543kb 40°C	Blanco et al., 1997

Table 6 is reproduced from Grosu et al. 2017 [under the Creative Commons Attribution License/public domain].

## Data Availability

All data analyzed or generated during this study are included in this published article.
